# Soil microbial community structures are shaped by agricultural systems revealing little temporal variation

**DOI:** 10.1016/j.envres.2022.113915

**Published:** 2022-11

**Authors:** A. Fox, F. Widmer, A. Lüscher

**Affiliations:** a Forage Production and Grassland Systems, Agroscope, Reckenholzstrasse 191, CH-8046, Zürich, Switzerland; b Molecular Ecology, Agroscope, Reckenholzstrasse 191, CH-8046, Zürich, Switzerland

**Keywords:** Agricultural systems, Bacteria, Fungi, Temporal stability, Next-generation sequencing

## Abstract

Many studies in soil microbial ecology are undertaken with a single sampling event, with the influence of temporal progression rarely being considered. Under field conditions, soil samples were taken from different agricultural systems; a sown grassland to maize rotation (MC), an intensively managed permanent grassland (INT), as well as extensively managed permanent grasslands with high (EXT_HP), low to sufficient (EXT_LP) and deficient available P (EXT_DP), six times throughout the 2017 growing season. Thus, this study aimed to determine if any differences in soil microbiome structures between both sharply contrasting (MC – INT – EXT), slightly differing (EXT_HP – EXT_DP) and quite similar (EXT_HP – EXT_LP and EXT_LP – EXT_DP) agricultural systems persist through changing growth conditions within the growing season. For both fungal and bacterial community structure, the influence of agricultural system (CV = 0.256, P < 0.001 and CV = 0.145, P < 0.01, respectively) was much greater than that of temporal progression (√CV = 0.065 and 0.042, respectively, both *P* < 0.001). Importantly, nearly all agricultural systems persistently harbored significantly distinct fungal community structures across each of the six sampling events (all at least *P* < 0.05). There were not as many pairwise differences in bacterial community structure between the agricultural systems, but some did persist (MC and EXT_HP ∼ EXT_DP, all *P* < 0.001). Additionally, persistent indicator fungal OTUs (IndVal >0.7, *P* ≤ 0.05) associated to each agricultural system (except EXT_LP) were found in each of the six sampling events. These results highlight the temporal stability of pairwise differences in soil microbiome structures between established agricultural systems through changing plant growth conditions, even between those with a comparable management regime. This is a highly relevant finding in informing the sampling strategy of studies in soil microbial ecology as well as for designing efficient soil biodiversity monitoring systems.

## Highlights

• Agricultural system had a much greater influence on fungal and bacterial community structure than that of temporal progression.
• Persistent pairwise differences in fungal community structure seen between sharply contrasting, slightly contrasting and similar systems.
• Persistent fungal indicator OTUs, associated to most of the studied agricultural systems, were found in each of the six sampling events.
• There were not as many persistent pairwise differences in bacterial community structures seen between agricultural systems.

## 1 Introduction

The soil microbiome is fundamental in a number of agroecosystem processes such as soil nutrient cycling, organic matter decomposition and plant productivity (Bertola et al., 2021). Agricultural management intensity is a major driver of soil microbiome community structure, with this influence having been observed at the plot (Hartmann et al., 2014), regional (Felipe-Lucia et al., 2020), continental (Fox et al., 2021), and even global scale (Leff et al., 2015). Soil microorganisms can, however, also exhibit temporal variation (Hannula et al., 2019), and the importance of such temporal aspects into soil microbiome studies is being increasingly highlighted in the literature (Geisen, 2021). Despite this, the extent of this influence on the structure of the soil microbial community in agricultural systems, as well as the stability of any differences in community structure between differently managed systems, remains poorly understood (Dunn et al., 2021). This is a pertinent knowledge gap, and has potentially profound implications on the sampling strategy of studies relating to soil microbiology and for designing efficient monitoring systems for soil biodiversity.

Managed grassland production systems undergo significant changes within a growing season, both in terms of agricultural management, plant phenological stage and weather conditions all of which are known to shape the structure of the soil microbiome community. Such systems, for example, undergo numerous utilizations in a single growing season (i.e., grazing and cuts) and can receive considerable fertilizer additions (Dindová et al., 2019). An even more drastic disturbance can be seen with crop rotations utilizing a sown grassland (a ley farming system; Lemaire et al., 2015). Here, a previous sown grassland sward is totally removed and the soil ploughed to create a seed bed for a following arable crop. In addition to these management practices, plant phenological development throughout the growing season will also influence soil microbial community structure (Bonkowski et al., 2021), as the plant's rooting system and patterns of rhizodeposition change. Lastly, variations in weather in a growing season may also determine the plant productivity of such systems, such as lower temperature and radiation towards the latter months and reduced rainfall in summer (Murphy et al., 2007). Erratic weather events may also occur, such as drought (Moravec, 2021), or heavy precipitation events (Padilla et al., 2019). As there is a close interconnection between plants and the soil microbiome, any environmental perturbation which affects the plant can also be expected to affect soil microbial community structure (Hartman and Tringe, 2019).

There are further indications that the temporal variation in soil microbial communities within the grassland land-use type or within the same management system in the short-term is quite considerable. For example, in an intensively managed grassland, it was shown that seasonal progression was a stronger driver of soil microbiome structure (measured using a DNA fingerprinting approach) than a single application of slurry (Fox et al., 2017). It has also been demonstrated that in intensively managed grasslands, soil microbial community structure (measured using phospholipid fatty acid analysis) was largely insensitive to inorganic phosphorus (P) fertilizer application, but responded strongly to seasonal progression (Massey et al., 2016). Along a gradient from an intensive to low-input grassland management, measures of both microbial biomass and the abundance of individual microbial fatty acids did show strong seasonal patterns, being at their maximum in spring and minimum in autumn (Bardgett et al., 1999). More recently, in a rarely managed permanent grassland, considerable temporal oscillations in the soil bacterial community were detected through April to November (Richter-Heitmann et al., 2020). The consequence of this temporal variation on the stability of differences in microbial community structure between more or less distinct grassland management types throughout the growing season does, however, remain unclear.

As a consequence, there is little consensus in the literature on when in the growing season to sample, nor how often in the season to sample, to best capture differences in soil microbial community structure between differently managed agricultural systems. Given that the vast majority of studies in soil microbial ecology undertaken have utilized a single sampling event, this urgently needs to be addressed. In light of this, the present study aimed to investigate the temporal development of differences in soil microbial community structures between both (i) sharply contrasting (ii) slightly differing and (iii) quite similar agricultural systems over the growing season.

Specifically, the study addressed three principle research hypotheses:

1) That sharply contrasting (sown grassland to maize rotation – intensive permanent grassland – extensive permanent grassland), slightly differing (extensive permanent grassland with high available P – extensive permanent grassland with deficient available P) and quite similar agricultural systems (e.g. extensive permanent grassland with low available P – extensive permanent grassland with deficient available P) harbor significantly distinct soil microbiome structures.
2) Given differences in management and weather conditions throughout the growing season, temporal effects on the soil microbiome could be expected.
3) Despite any temporal changes, differences in microbiome structures between the different agricultural systems persist and are consistently detectable.

## 2 Materials and methods

### 2.1 Study design and soil sampling

Five different agricultural systems were sampled (five independent fields per system, yielding twenty-five study sites in total), which varied in their management intensity (i.e., number of yearly utilizations and nutrient inputs) and soil P availability. The most intensive system sampled was a sown temporary grassland (∼2 years since establishment) converted to maize (MC, abbreviation for ‘maize crop’). Four permanent grassland types were also sampled, comprising intensively (INT, i.e., high fertilization rate, early and frequent utilizations) managed grasslands, as well as extensively managed, unfertilized grasslands with high (EXT_HP), low to sufficient (EXT_LP) and deficient (EXT_DP) levels of available soil phosphorus. The fifteen extensive sites were grouped into the five sites with the highest available P, the five sites with the lowest available P, while the remaining five sites were considered the intermediate group. The mean value of available P for each group was then interpreted based on the threshold for available P based on Swiss agronomic advice (Agroscope, 2017; Liebisch et al., 2013).

All twenty-five sites were sampled around the Zurich region of Switzerland (Fig. S1). The extensive grassland systems were classified ‘ecological compensation areas’ and thus the farmers received subsidies to manage these sites through infrequent utilizations and no fertilizer inputs. MC was sampled on account of the fact that soil ploughing represents a major soil disturbance, in comparison to the relative stability of the permanent grassland systems. The purpose of this sampling strategy was to investigate the temporal persistence of any differences in soil microbial community structures between both sharply contrasting (MC – INT – EXT), slightly differing (EXT_HP – EXT_DP) and quite similar (EXT_HP – EXT_LP and EXT_LP – EXT_DP) agricultural systems. Here, the strategy in relation to the grouping of the EXT sites was particularly appropriate, as both EXT_HP and EXT_DP differed significantly in terms of their available P and pH (Table 1), but both values from each of these systems were so near to EXT_LP that they differed only numerically, not statistically. Additionally, the ploughing event in the MC system was specifically taken to test the efficacy of our sampling strategy to detect changes in microbial community composition due to this significant disturbance.

**Table 1:** The mean values of various soil physicochemical and climatic parameters (mean ± SE) in each agricultural system; sown grassland to maize rotation (MC), intensive grassland (INT), extensive grassland with high, low and deficient soil phosphorus levels (EXT_HP, EXT_LP and EXT_DP, respectively, the five categories are collectively referred to as ‘systems’). Significance code: ‘***’ *P ≤* 0.001 ‘**’ *P ≤* 0.01 ‘*’ *P ≤* 0.05 ‘ns*’ P >* 0.05. Pairwise differences between systems is shown through different letters. Shown is the difference between the systems in terms of soil pH_H2O_ (pH), total % soil C (C_tot_) and N (N_tot_), total (P_tot_) and available (P_av_) soil P (mg/g) and the % soil clay (Clay), sand (Sand) and silt (Silt). Also shown is the climatic variables mean annual temperature (mean_T, °C), mean annual rainfall (mean_RF, mm) and altitude (Altitude, m).

	F value	MC	INT	EXT_HP	EXT_LP	EXT_DP
pH	4.652**	6.64 (±0.186)^ab^	6.98 (±0.177)^ab^	7.40 (±0.144)^a^	7.00 (±0.290)^ab^	6.13 (±0.270)^b^
C_tot_	3.943*	2.91 (±0.377)^b^	5.14 (±0.434)^a^	4.71 (±0.380)^ab^	4.08 (±0.771)^ab^	3.05 (±0.414)^ab^
N_tot_	3.293*	0.27 (±0.039)^b^	0.45 (±0.046)^a^	0.38 (±0.052)^ab^	0.32 (±0.022)^ab^	0.29 (±0.042)^ab^
P_tot_	4.809**	98.73 (±13.829)^ab^	120.75 (±15.030)^a^	94.52 (±9.753)^ab^	70.73 (±4.465)^b^	58.62 (±9.430)^b^
P_av_	25.76***	15.02 (±2.235)^a^	16.51 (±0.892)^a^	8.92 (±1.557)^b^	4.46 (±0.142)^bc^	1.43 (±0.155)^c^
Clay	1.877^ns^	21.61 (±1.320)	20.89 (±2.104)	22.05 (±1.835)	27.92 (±1.151)	25.35 (±3.567)
Sand	1.359^ns^	29.34 (±1.174)	37.25 (±3.897)	34.22 (±3.712)	27.61 (±0.847)	33.39 (±4.919)
Silt	1.496^ns^	49.05 (±1.886)	41.86 (±2.340)	43.75 (±3.619)	44.47 (±1.491)	41.26 (±2.696)
mean_T	2.152^ns^	9.62 (±0.115)	9.48 (±0.155)	9.33 (±0.086)	9.24 (±0.101)	9.28 (±0.054)
mean_RF	2.933*	1169 (±28.5)^a^	1140 (±31.7)^a^	1092 (±21.3)^a^	1089 (±8.1)^a^	1085 (±6.3)^a^
Altitude	0.134^ns^	503 (±22.4)	486 (±32.7)	493 (±13.8)	505 (±16.9)	499 (±11.5)

A management questionnaire was sent to each of the participating farmers, asking to provide information as to the rate of fertilizer application, number of cuts and grazing intensity on their farm. Both INT and MC received N fertilizer input,in mineral (ammonium nitrate) or organic form (or both), at rates of 136.4 and 213.7 kg N ha^−1^ yr^−1^. None of the extensive grassland systems received any nutrient fertilization. Additionally, each of the extensively managed grasslands were just used for cuttings (avg. 2 cuts yr^−1^) with none of them being grazed. The management of INT was more mixed, with the sites being both cut and grazed, with a high average grazing intensity (1082 livestock units (LU) days ha^−1^ yr^−1^). Prior to the planting of the maize crop for MC, the sown grassland sward was cut, though on one site it was lightly grazed (50 LU day ha^−1^ yr^−1^).

### 2.2 Soil sampling and preparation

Soil samples were taken at each site six times throughout the length of the 2017 grassland growing season; 10^th^ May (1), 6^th^ June (2), 27^th^ June (3), 15^th^ August (4), 12^th^ September (5) and 9^th^ October (6). The selection of these samplings times were to coincide with inevitable weather and seasonal changes associated with temporal progression, but also with specific management practices. These were namely (i) before and after the ploughing and sowing of maize (samplings 1 and 2, respectively), (ii) before and after the harvesting of the extensive grasslands (samplings 2 and 3), as well as (iii) before and after the harvesting of maize (samplings 5 and 6). A graphical overview of both the study and sampling strategies is provided in Fig. S2.

At each sampling date, sixteen soil cores were taken in the center of the site (two straight lines of eight cores, approx. two meters apart) using an auger (ø 2.5 cm) to a depth of 20 cm. Field edges were avoided to a distance of 10 m. The sixteen cores were then combined, and fresh soil was homogenized through a 2 mm sieve. Approximately 0.5 g (fresh weight) of each sample was then added to a 2 ml Eppendorf tube containing 0.5 g of glass beads (ø 0.10–0.11 mm). 1.2 ml extraction buffer (0.2 M Na_3_PO_4_ of pH 8, 0.1 M NaCl, 50 mM EDTA, 0.2% CTAB) was added, and the tubes were vortexed and stored at −20 °C until DNA extraction (Fox et al., 2020).

### 2.3 Measurement and modelling of environmental variables

The remaining >2 mm soil (after the 0.5 g for DNA extraction was taken) was used to determine soil characteristics. To determine total soil P content (P_tot_), 300 mg of air-dried, milled soil was burned (550 °C, 3 h, Nabertherm B400) to remove the organic matter and the remaining ash was digested in 10 ml 13% HNO_3_ (1:4) and analyzed using ICP-OES. Available soil P content (P_av_) was determined calorimetrically after extracting in ammonium lactate and acetic acid (Egner et al., 1960). Total soil carbon (C_tot_) and nitrogen (N_tot_) was measured using the FLASH 2000 Elemental analyzer (Thermo Scientific, Waltham, MA). Soil pH was determined in a 1:5 ddH_2_O suspension. Soil texture (i.e., the relative amounts of sand, silt and clay) was quantified using the particle fractionation method, combining sieving and sedimentation (ISO 11277:2009). Daily data for precipitation, mean, minimum and maximum temperature covering 1981–2017 were extracted from the Spatial Climate Analyses provided by the Federal Office of Meteorology and Climatology (MeteoSwiss). The climate analyses has a spatial resolution of 2 km × 2 km, and we considered the nearest grid points as representative for each site location. Monthly statistics for temperature were corrected for the difference in altitude between site and nearest grid point. To do so, 4th-degree polynomials representing the altitudinal change in temperature were fitted, for each month individually, to the monthly climatic norms 1981–2010, which were used from a total of 89 weather stations.

### 2.4 DNA extraction and metabarcoding

Soil DNA was extracted from each sample in three repeated extractions according to the procedure outlined previously (Bürgmann et al., 2001), in addition to the modifications described by (Hartmann et al., 2005). DNA extracts were quantified using the PicoGreen dsDNA quantification assay (Invitrogen) using a Cary Eclipse fluorescence spectrophotometer (Varian Inc., Palo Alto, CA), and then diluted with sterile ddH_2_O to a concentration of 5 ng/μl. The fungal internal transcribed spacer region (ITS2) of the rRNA operon was PCR amplified by using primers ITS3 (5′ CAH CGA TGA AGA ACG YRG 3′) and ITS4 (5′ TCC TSC GCT TAT TGA TAT GC 3′, Tedersoo et al., 2014), while the primer pair 341F (5′ CCT AYG GGD BGC 216 WSC AG 3′) and 806R (5′ GGA CTA CNV GGG THT CTA AT 3′) was used to PCR amplify the V3–V4 region of the bacterial 16S rRNA gene (Frey et al., 2016). The 5’ end of each forward primer was tagged with a CS1 adapter, while the reverse primer was tagged with CS2, which allowed multiplexing with the Fluidigm Access Array System (Fluidigm, San Francisco, CA). PCR amplification conditions were as previously described (Mayerhofer et al., 2017), with the modification that reaction volumes of 20 μl and 35 amplification cycles were used. PCR amplification was performed for each sample in four separate reactions, which were pooled and sent to the Génome Québec Innovation Center for PE300 sequencing using the Illumina Miseq v3 platform (Illumina Inc. San Diego, CA).

### 2.5 Sequence processing, taxonomic classification and alpha diversity estimation

Sequences were processed through a customized pipeline in UPARSE implemented in USEARCH version 9 (Edgar, 2010) with the specific details described previously (Frey et al., 2016; Mayerhofer et al., 2017). Taxonomic classification of operational taxonomic unit (OTU) centroids (clustered at 97% identity) was performed using the Bayesian classifier implemented in MOTHUR version 1.36.1 (Schloss et al., 2009). The SILVA ribosomal RNA gene database (version 132, Quast et al., 2012) was used as a reference for prokaryotic sequences, with prokaryotic OTUs assigned to chloroplasts, mitochondria, and archaea being removed to retain only bacterial OTUs (bOTUs) for analysis. A custom-made database extracted from the NCBI GenBank (Benson et al., 2014) was used as a reference for eukaryotic sequences. Nonfungal sequences were discarded from the eukaryotic dataset, and retained fungal OTUs (fOTUs) were assigned using the UNITE reference database (version 7.2, Abarenkov et al., 2010). Fungal and bacterial OTU richness and the inverse Simpson index was calculated using the ‘summary.single’ command in the MOTHUR software. When calculating these measures, differences in sequencing depth were corrected for by an iterative (10,000x) subsampling to the lowest sequence number per sample in the dataset (23,265 sequences for fungi and 11,934 sequences for bacteria, Fig. S3).

### 2.6 Data analysis

Both the fungal and bacterial OTU tables were square-root transformed, converted to relative abundance and a pairwise Bray-Curtis dissimilarity matrix was constructed for each (Hartmann et al., 2012). This served as the response in a repeated measures PERMANOVA to determine the influence of agricultural system (factor ‘System’), sampling event (factor ‘Time’) and the ‘System × Time’ interaction on fungal community structure (PRIMER-7 statistical software package, version 7, Plymouth UK; Clarke and Warwock, 2001). Pairwise differences in community structures within and between the different agricultural systems across sampling events was also determined. A repeated measures analysis of variance (ANOVA) was used to determine the effect of the above-mentioned factors on fungal and bacterial community OTU richness and inverse Simpson measures.

Associations of OTUs to each agricultural system was determined by correlation based indicator species analysis (‘multiplatt’ function) with 9,999 permutations in the "indicspecies" package (Cáceres and Legendre, 2009). Indicator OTUs were defined as those with an IndVal ≥0.7 and a *P* ≤ 0.05.

## 3 Results

### 3.1 Strong effect of the agricultural systems on soil nutrient parameters

The soils selected in this study were of a similar soil type, thus there was no pairwise difference (*P* > 0.05) between the permanent grassland systems and MC in terms of their soil texture, in either their % clay (avg. 23.56%), sand (avg. 32.62%) or silt (avg. 44.08%). Likewise, there was no significant difference (*P* > 0.05) in either mean temperature (avg. 9.39 °C) or mean relative rainfall (avg. 1115 mm). The factor ‘System’, however, did have a significant effect on the soil nutrient values measured in this study (all results in this section are presented in Table 1); C (*P* < 0.05), N (*P* < 0.05), P_tot_ (*P* < 0.01) and P_av_ (*P* < 0.001). For both total soil C and N, the measured values were significantly higher in INT (5.14 and 0.45, respectively) when compared to MC (2.91, 0.27, respectively), but not from the other three grassland systems (*P* > 0.05). Both INT (16.51 mg/g) and MC (15.02 mg/g) had significantly higher levels of P_av_ when compared against each of the extensive grassland management systems, EXT_HP (8.92 mg/g), EXT_LP (4.46 mg/g) and EXT_DP (1.43 mg/g). EXT_HP had a significantly higher P_av_ than EXT_DP, though neither were significantly different to EXT_LP. Additionally, INT (120.75 mg/g) had significantly higher levels of P_tot_ when compared against both EXT_LP (70.73 mg/g) and EXT_DP (58.62 mg/g), but not from EXT_HP. There was also a significant effect of ‘System’ on soil pH (*P* < 0.01), with EXT_HP having a higher pH than EXT_DP.

### 3.2 Agricultural systems are stronger determinants of soil microbiome structures than temporal progression during the growing season

A total of 6,784,558 high-quality partial fungal ITS sequences were obtained with a mean Good's coverage per sample of 1, yielding 8,418 fOTUs. The factors included in the repeated measures PERMANOVA model; both ‘System’ and ‘Time’ had a highly significant effect on fungal community structure, as well as there being a highly significant ‘System × Time’ interaction (all *P* < 0.001, Table 2a). When looking at the square-root of the component of variation of the model, it became clear that the factor ‘System’ (√CV = 0.256) had the far stronger influence on fungal community structure than either the factor ‘Time’ (√CV = 0.065) or the ‘System × Time’ interaction (√CV = 0.056, Table 2a).

**Table 2:** Results of a repeated measures PERMANOVA model of the effect of agricultural system (‘System’), temporal progression (‘Time’), Replicate (System) denotes the term for the replicate sites over which the factor ‘System’ is tested, and the ‘System × Time’ interaction on both the fungal (a) and bacterial (b) community structure. Shown is the degrees of freedom (df), the mean sum of squares (MS), the Pseudo –F value and the square-root of the component of variation (√CV). Significance code: ‘***’ *P ≤* 0.001 ‘**’ *P ≤* 0.01 ‘*’ *P ≤* 0.05.

	Factor	df	MS	Pseudo F	√CV
(a) Fungi					
System		4	2.647	3.878***	0.256
Rep (System)		20	0.683		0.319
Time		5	0.178	2.456***	0.065
System × Time		20	0.088	1.214***	0.056
Residual		100	0.072		0.269
(b) Bacteria					
System		4	0.974	2.855**	0.145
Rep (System)		20	0.341		0.219
Time		5	0.100	1.823***	0.042
System × Time		20	0.060	1.093*	0.032
Residual		100	0.055		0.234

For bacteria, 3,303,574 high quality partial 16S rRNA gene sequences were obtained (mean Good's coverage per sample 0.95), resulting in 15,391 bOTUs. Like with the fungi, both the factors ‘System’ and ‘Time’ had a highly significant effect on bacterial community structure (at least *P* < 0.01, Table 2b), as well as there being a significant ‘System × Time’ interaction (*P* < 0.05). The effect of ‘System’ (√CV = 0.145) was, however, much greater than that of ‘Time’ (√CV = 0.042) and the ‘System × Time’ interaction (√CV = 0.032).

### 3.3 Pairwise differences of fungal community structures between agricultural systems persist over the growing season

**Fig. 1:**
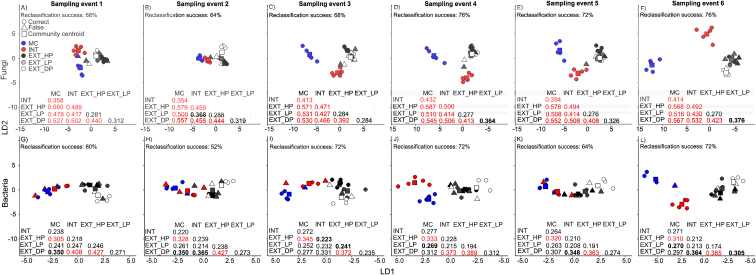
Differentiation of both fungal (A–F) and bacterial (G–L) community structure between the five agricultural systems (MC, INT, EXT_HP, EXP_LP & EXT_DP) for each of the six sampling events (1–6). Circles indicate soil samples which were correctly reclassified, while triangles indicate samples which were incorrectly reclassified to its sampled agricultural system in a leave-one-out reclassification test. Reclassification and constrained ordination are based on canonical analyses of principal coordinates (CAP) when maximizing differences between the five agricultural systems. Axes show linear discriminants (LD). Also displayed in each panel is the community centroid distance (Euclidian distance) between each of the agricultural systems, with statistically significant differences being indicated as: ‘red’ *P ≤* 0.001 ‘bold’ *P ≤* 0.05 and ‘unbold’ *P >* 0.05. Squares represent the community centroids for each of the five agricultural systems. Explanations for abbreviations can be found in Table 1.

When looking at the pairwise differences in fungal community structure between the agricultural systems at each single sampling event, it became evident that the fungal community structure in INT differed highly significantly from each of the extensively managed grassland systems; EXT_HP, EXT_LP and EXT_DP (avg. centroid distance = 0.469, all *P* < 0.001, Fig. 1A) at the first sampling event. Remarkably, it was also highly significantly different from MC when, at this sampling event, this treatment was still an intensively managed sown grassland (centroid distance = 0.358, *P* < 0.001, Fig. 1A).

Interestingly, the significant difference in fungal community structure between INT and each of the permanent extensive grassland systems persisted throughout the five following sampling events of the growing season (Fig. 1B–F, centroid distance range of 0.368–0.532, in most cases *P* < 0.001). The highly significant difference between INT and MC observed for the first sampling also persisted through all other sampling events, when the sown grassland was ploughed and the maize crop planted (Fig. 1B–F, centroid distance range = 0.354–0.432, all *P* < 0.001). The fungal community structure of MC was also highly significantly different from each of the extensive permanent grassland systems at the first sampling event (Fig. 1A, centroid distance range = 0.478–0.560, all *P* < 0.001), and remained so throughout the remainder of the growing season (Fig. 1B–F, centroid distance range = 0.500–0.587, all *P* < 0.001).

For the extensive grasslands, EXT_HP and EXT_DP had a highly significantly distinct fungal community structure at sampling event 1 (centroid distance = 0.440, *P* < 0.001), which persisted throughout all subsequent sampling events (Fig. 1B–F, centroid distance range = 0.392–0.444, all *P* < 0.001). Neither EXT_HP nor EXT_DP had significantly distinct fungal community structures compared to EXT_LP at the first sampling event (Fig. 1A, *P* > 0.05). For EXT_HP, this non-significance persisted across all subsequent sampling events (*P* > 0.05, Fig. 1B–F). This was also the case for EXT_DP at sampling events 2, 3 and 5 (*P* > 0.05), but there was a significant difference in fungal community structure between EXT_LP and EXT_DP at the sampling events 4 (Fig. 1D, centroid distance range = 0.364, *P* < 0.05) and 6 (Fig. 1F, centroid distance range = 0.376, *P* < 0.01).

**Fig. 2:**
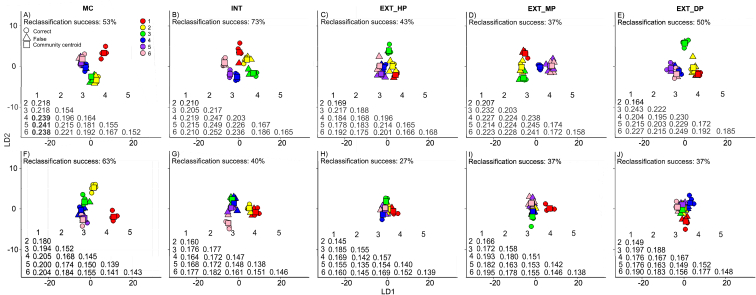
Differentiation of both fungal (A–E) and bacterial (F–J) community structure between the six sampling events (1–6) for each of the five agricultural systems (MC, INT, EXT_HP, EXP_LP & EXT_DP). Circles indicate soil samples which were correctly reclassified, while triangles indicate samples which were incorrectly reclassified to its sampled agricultural system in a leave-one-out reclassification test. Reclassification and constrained ordination are based on canonical analyses of principal coordinates (CAP) when maximizing differences between the six sampling events. Axes show linear discriminants (LD). Also displayed in each panel is the community centroid distance (Euclidian distance) between each of the sampling events, with statistically significant differences being indicated as: ‘bold’ *P ≤* 0.05 and ‘unbold’ *P >* 0.05. Squares represent the community centroids for each of the six sampling events within each system. Explanations for abbreviations can be found in Table 1.

The pairwise differences in fungal community structure within agricultural systems across each sampling event was also examined. Here, it was seen that within each of the permanent grassland systems, INT, EXT_HP, EXT_LP, EXT_DP, there was no significant difference between any of the six sampling events (Fig. 2B–E, *P* > 0.05). Contrastingly, the fungal community structure within MC was significantly different (*P* < 0.05) between sampling event 1, when it was a sown grassland, and sampling events 4 (Fig. 2A, centroid distance = 0.239), 5 (Fig. 2A, centroid distance = 0.241) and 6 (Fig. 2A, centroid distance = 0.238), when the maize crop had become established.

**Fig. 3:**
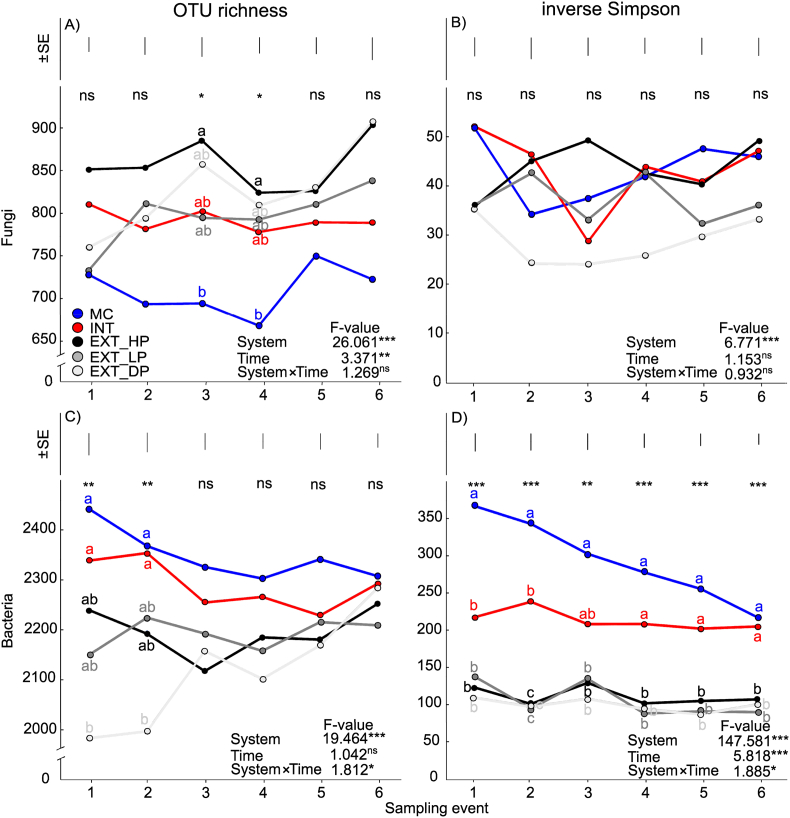
The mean values for fungal community OTU richness (A), inverse Simpson index (B) as well as the bacterial community OTU richness (C) and the inverse Simpson index (D) in the five sampled agricultural systems across the six sampling events. Also shown (as an insert in each panel) are the F-values from the repeated measures ANOVA testing the effect of the factors ‘System’ and ‘Time’, as well as the ‘System × Time’ interaction for each variables. The statistical significance for each is also indicated, Significance code: ‘***’ *P ≤* 0.001 ‘**’ *P ≤* 0.01 ‘*’ *P ≤* 0.05 and ‘ns’ *P >* 0.05. The effect of management in each sampling period is indicated using the same statistical code at top of each panel, with the significant pairwise differences between systems at each sampling event indicated by different letters. Lastly the variation (±SE) at each sampling event is indicated by an error bar at the top of each panel. Explanations for abbreviations can be found in Table 1.

Looking at fungal alpha diversity, there was a highly significant effect of the factor ‘System’ (*F* = 26.061, *P* < 0.001) on fungal OTU richness. The factor ‘Time’ also had a significant effect on fungal OTU richness (*P* < 0.01), though it had a far lower effect size than the factor ‘System’ (*F* = 3.372). There was no ‘System × Time’ interaction on fungal OTU richness (*F* = 1.269, *P* > 0.05, Fig. 3A). The highest fungal OTU richness (avg. across all of the six sampling events) was found in EXT_HP (857 OTUs) and the lowest was in MC (708 OTUs). Visualizing fungal OTU richness across the six individual sampling events, while temporal variation was seen, there was little overlap in the values between the different agricultural systems, particularly between MC, INT and EXT_HP (Fig. 3A). There was also a highly significant effect of the factor ‘System’ on the fungal community inverse Simpson index (*F* = 6.771, *P* < 0.001), though there was no effect of ‘Time’ nor a ‘System × ‘Time’ interaction (*F* = 1.153 and *F* = 0.932, respectively, both *P* > 0.05, Fig. 3B). The highest fungal community inverse Simpson index value (avg. across all of the six sampling events) was found in both MC and INT (43) and the lowest was in EXT_DP (29).

### 3.4 Less persistent pairwise differences between systems in terms of bacterial community structure

When looking at the pairwise differences in bacterial community structure, INT had a highly significantly different bacterial community structure compared to EXT_DP (Fig. 1G, centroid distance = 0.408, *P* < 0.001) at the first sampling event, but not from the other two extensive grasslands or from MC (Fig. 1G, *P* > 0.05). Both EXT_HP (Fig. 1G, centroid distance = 0.427, *P* < 0.001) and MC (Fig. 1G, centroid distance = 0.350, *P* = 0.026) also had significantly distinct bacterial community structures compared to EXT_DP, as well as from each other at this sampling event (Fig. 1G, centroid distance = 0.305, *P* < 0.001). The only two pairwise differences in bacterial community structure to persist throughout the following sampling events were the differences between EXT_HP and EXT_DP (Fig. 1H–L, centroid distance of 0.363–0.427, all *P* < 0.001) and between EXT_HP and MC (Fig. 1H–L, centroid distance of 0.310–0.345, all *P* < 0.001). The pairwise differences in bacterial community structure between INT and EXP_DP also mostly remained significant over the following sampling events (Fig. 1H–L, centroid distance = 0.331–0.373, mostly *P* < 0.05), but just failed to reach significance at sampling event 3 (*P* = 0.057). In contrast, the significant difference between MC and EXT_DP seen at sampling event 1 did persist into sampling event 2 (Fig. 1H, centroid distance = 0.350, *P* < 0.01), but differences in bacterial community structures between the two did not reach significance at any of the later sampling events (Fig. 1I-L, *P* > 0.05). There were no pairwise differences in bacterial community structure within the different systems throughout the six sampling events of the study (Fig. 2F–J, all *P* > 0.05).

Looking at bacterial OTU richness, the factor ‘System’ was the strongest explanatory variable (*F* = 19.143, *P* < 0.001). There was no significant effect of the factor ‘Time’ (*P* > 0.05), though there was a significant ‘System × Time’ interaction (*P* < 0.05, Fig. 3C). Bacterial OTU richness was significantly higher in both INT (avg. across the six sampling events 2289 OTUs) and MC (avg. 2348 OTUs) than in any of the three extensive permanent grassland systems (range avg. 2115–2194 OTUs). The factor ‘System’ was also by the far the strongest determinant of the bacterial community inverse Simpson index (*F* = 147.581, *P* < 0.001). For this variable, both the factor ‘Time’ and the ‘System × Time’ interaction had a highly significant effect (*F* = 5.818 and *F* = 1.885, respectively, both at least *P* < 0.05, Fig. 3D). When visualizing the bacterial community inverse Simpson index across the six sampling events, there was minimal overlap between the different agricultural systems, despite temporal variation, particularly between MC, INT and the EXT systems (Fig. 3D).

### 3.5 Agricultural system associated indicator OTUs detected in multiple sampling events

There was a persistence of fungal indicator OTUs (fOTUs) across the sampling events (Table 3a), with some being detected at all sampling events for each system, except for EXT_LP. Five of these were associated to INT, three each for EXT_HP and EXT_DP and eleven to MC (Table 3a). Some of the taxonomic assignments of the fOTUs associated to the extensive grasslands were to the family Clavariaceae (fOTU_70, EXT_HP) and the species *Pezizomycetes_sp* (fOTU_408, EXT_HP), and *Orbiliomycetes_sp* (fOTU_398 & 433, EXT_DP). For INT and MC, some of the indicator fOTUs were taxonomically assigned to the genera; *Podospora* (fOTU_837, INT), *Chaetomium* (fOTU_227, MC), *Exophiala* (fOTU_2777, MC), *Talaromyces* (fOTU_5876, MC) and to the species; *Helotiales* (OTUf_153, MC) and *Plectosphaerella_cucumerina* (OTUf_6397, MC, Table 3a). Furthermore, there were 37 system indicator fOTUs identified that persisted over five sampling events and 49 over four sampling events (Table S2). Most of these fOTUs (14 and 16, respectively) were associated to MC at five and four sampling events. Many of these indicator fOTUs had a strong association with their respective system (i.e., IndVal >0.9). The effect of the factors ‘System’, ‘Time’ and the ‘System × Time’ interaction on the major fungal phyla (i.e., those with a % relative abundance greater than 0.1%) is shown in the supplementary materials (Table S1a).

**Table 3:** Fungal (a) and bacterial (b) OTUs (fOTUs and bOTUs, respectively, mean ± SE) which were detected as unique indicator OTUs (IndVal ≥0.7, *P* ≤ 0.05) at each of the six sampling events (1–6) for each of the five systems (MC, INT, EXT_HP, EXT_LP and EXT_DP) through indicator species analysis. Shown here is the average IndVal and *P* -value over the six sampling events. Taxonomy of each detected OTU is given at the lowest known taxonomic level: k_: Kingdom, p_: Phylum, c_: Class, o_: Order, f_: Family, g_: genus, s_: species. Significance, code: ‘**’ *P ≤* 0.01 ‘*’ *P ≤* 0.05.

OTU	System	Taxonomy	IndVal	MC	INT	EXT_HP	EXT_LP	EXT_DP
(a) Fungi								
OTUf_1154	MC	k__Fungi	0.920**	0.021 (±0.0033)	0.002 (±0.0010)	0	<0.001 (±0.0003)	<0.001 (±0.0003)
OTUf_1332	MC	k__Fungi	0.833**	0.023 (±0.0063)	0.002 (±0.0006)	0	0.001 (±0.0007)	<0.001 (±0.0001)
OTUf_153	MC	s__Helotiales_sp	0.873**	0.317 (±0.0971)	0.005 (±0.0045)	<0.001 (±0.0002)	<0.001 (±0.0002)	<0.001 (±0.0002)
OTUf_227	MC	g__Chaetomium	0.964**	0.472 (±0.1038)	0.007 (±0.0045)	0.003 (±0.0012)	0.006 (±0.0029)	0.002 (±0.0014)
OTUf_2777	MC	g__Exophiala	0.887**	0.296 (±0.0862)	0.006 (±0.0062)	<0.001 (±0.0001)	0.001 (±0.0005)	<0.001 (±0.0003)
OTUf_379	MC	c__Sordariomycetes	0.808*	0.104 (±0.0539)	0.001 (±0.0012)	0	<0.001 (±0.0001)	0
OTUf_4347	MC	o__Pleosporales	0.884**	0.053 (±0.0140)	0.002 (±0.0012)	0.001 (±0.0003)	0.002 (±0.0010)	0.001 (±0.0007)
OTUf_450	MC	s__Sordariomycetes_sp	0.833*	0.086 (±0.0179)	0.003 (±0.0027)	0	<0.001 (±0.0003)	<0.001 (±0.0001)
OTUf_586	MC	s__Microascus_verrucosus	0.905**	0.105 (±0.0367)	0.004 (±0.0014)	<0.001 (±0.0002)	0.001 (±0.0004)	<0.001 (±0.0001)
OTUf_5876	MC	g__Talaromyces	0.879**	0.271 (±0.0512)	0.006 (±0.0058)	0.012 (±0.0103)	0.001 (±0.0003)	<0.001 (±0.0004)
OTUf_6397	MC	s__Plectosphaerella_cucumerina	0.934**	0.135 (±0.0202)	0.007 (±0.0061)	<0.001 (±0.0001)	0.003 (±0.0013)	<0.001 (±0.0001)
OTUf_437	INT	o__Pleosporales	0.869**	0.014 (±0.0055)	0.065 (±0.0099)	0.001 (±0.0005)	0.002 (±0.0015)	<0.001 (±0.0001)
OTUf_493	INT	s__Preussia_persica	0.929**	0.002 (±0.0009)	0.116 (±0.0152)	0.002 (±0.0006)	0.002 (±0.0008)	0.006 (±0.0034)
OTUf_558	INT	o__Sordariales	0.924**	<0.001 (±0.0001)	0.058 (±0.0131)	0	0	0
OTUf_837	INT	g__Podospora	0.861*	<.001 (±0.0003)	0.045 (±0.0135)	0	0.001 (±0.0001)	<0.001 (±0.0002)
OTUf_905	INT	o__Pezizales	0.847*	0	0.036 (±0.0072)	0.001 (±0.0005)	0.004 (±0.0027)	<0.001 (±0.0001)
OTUf_408	EXT_HP	s__Pezizomycetes_sp	0.969**	0.001 (±0.0006)	<0.001 (±0.0001)	0.331 (±0.0717)	0.008 (±0.0063)	0.001 (±0.0007)
OTUf_552	EXT_HP	p__Ascomycota	0.873**	0	0	0.078 (±0.0354)	<0.001 (±0.0001)	<0.001 (±0.0001)
OTUf_703	EXT_HP	f__Clavariaceae	0.864**	0	0	0.055 (±0.0123)	0.002 (±0.0011)	<0.001 (±0.0003)
OTUf_398	EXT_DP	s__Orbiliomycetes_sp	0.849*	0	<0.001 (±0.0001)	0.001 (±0.0006)	0.014 (±0.0064)	0.125 (±0.0250)
OTUf_433	EXT_DP	s__Orbiliomycetes_sp	0.843*	0	0.001 (±0.0008)	0.001 (±0.0005)	0.008 (±0.0046)	0.107 (±0.0209)
OTUf_871	EXT_DP	p__Ascomycota	0.792*	0	0	<0.001 (±0.0001)	0.004 (±0.0028)	0.077 (±0.0167)
(b) Bacteria								
OTUb_1947	EXT_DP	f_Pedosphaeraceae	0.811*	<0.001 (±0.0001)	0.001 (±0.0003)	0.001 (±0.0003)	0.001 (±0.0009)	0.022 (±0.0039)
OTUb_2168	EXT_DP	g_Pajaroellobacter	0.760*	0	0	<0.001 (±0.0002)	0.003 (±0.0012)	0.019 (±0.0037)

There were much less persistent bacterial indicator OTUs (bOTUs) than fungal OTUs associated to each agricultural system. Just two indicator bOTUs (bOTU_1947 and bOTU_2168), associated to EXT_DP, were found in each of the six sampling events throughout the growing season (Table 3b). In addition, 22 system indicator bOTUs were identified to persist at five sampling events and 45 at four sampling events (Table S3). Most of these (8 and 19) were associated to MC at five and four sampling events, respectively. The effect of the factors ‘System’, ‘Time’ and the ‘System × Time’ interaction on the major bacterial phyla (i.e., those with a % relative abundance greater than 0.1%) is shown in the supplementary materials (Table S1b).

## 4 Discussion

### 4.1 Agricultural systems are a strong and temporally persistent driver of fungal community structures

The principle finding of this study is that the overwhelming driver of the soil fungal community structure is agricultural system, independent of the sampling time in the growing season. This is evident from the effect sizes of the factor ‘System’ on both fungal and bacterial community structure (√CV) and from the very stable distances (and their significance levels) among systems over all the sampling events. While there was a highly significant effect of the factor ‘Time’ on soil fungal community structure (√CV = 0.065), as well as a highly significant ‘System × Time’ interaction (√CV = 0.056), the influence of the factor ‘System’ (√CV = 0.256) was still evident and far greater. This finding is in agreement with the first hypothesis of this study that sharply contrasting (MC – INT – EXT), slightly differing (EXT_HP – EXT_DP) and quite similar (EXT_HP – EXT_LP and EXT_LP – EXT_DP) agricultural systems would differ in terms of fungal community structure. A strong effect of grassland management on soil microbial community structures has been demonstrated previously (Sayer et al., 2013; Szukics et al., 2019), with it even being shown to have a comparable effect on fungal community structure to continental-scale geographic factors (Barreiro et al., 2022; Fox et al., 2021). All of these studies, however, utilized only a single sampling event.

While a stronger effect of land-use on soil fungal community structure, compared to temporal progression, has been demonstrated previously (Gschwend et al., 2021a; Lauber et al., 2013), these studies have solely used sharply contrasting systems (e.g. grassland vs. arable). A stronger effect of tillage and cropping system, compared to sampling events (which ranged from pre-planting to crop maturity) has also recently been reported on soil fungal community structure (Wang et al., 2021). In this study, we demonstrate this effect using both sharply contrasting (MC – INT – EXT), slightly differing (EXT_HP – EXT_DP) and quite similar (EXT_HP – EXT_LP and EXT_LP – EXT_DP) agricultural systems, and this effect was not only seen in terms of soil fungal community structure. A stronger effect of the factor ‘System’, in comparison to the factor ‘Time’ was also observed in terms of fungal OTU richness (*F*-value = 26.061 and 3.372, respectively) and iS (*F*-value = 6.771 and 1.153, respectively). This finding is in line with that reported in cultivated corn and prairie systems in soil aggregate-size fractions (Upton et al., 2019). Such management induced differences reflect the differences in soil nutrient status between the studied systems. There was a significant influence of agricultural system on many of the measured soil physicochemical parameters, pH, % C and % N, total and available P, reflecting the varying intensity of management employed. All, except soil pH, were highest in INT, which emphasizes that these grasslands were good representations of intensively managed permanent grasslands (Wesche et al., 2012).

In agreement with our third hypothesis, we did show that differences in fungal community structure detected between the different agricultural systems at the start of the growing season did persist as the season progressed. There were significant differences between each agricultural system at all sampling events, with the exception of the “intermediate” EXT_LP from the other two extensive grassland systems. Considerable temporal variation in differences in soil microbial community structures between very similar/the same grassland management systems have been previously reported (Fox et al., 2017; Massey et al., 2016; Richter-Heitmann et al., 2020). Our results indicate that while temporal progression does influence soil fungal community structure (the effect of the factor ‘Time’ was highly significant, *P* < 0.001), it does not obfuscate the differences in said structures between managed grassland systems, even when they have the same management, but only differ in terms of their available P (i.e., EXT_HP vs. EXT_DP).

The temporal stability of soil fungal community structure was further highlighted by the lack of within agricultural system differences seen throughout the growing season. We saw that there was no significant difference in fungal community structure from sampling events 1–6 in the permanent grassland systems (INT, EXT_HP, EXT_LP and EXT_DP). Only in MC was there a significant shift in soil fungal community structure, with sampling events 4, 5 and 6 being significantly different from sampling event 1. This is most likely indicative of the change from a sown grassland to an established arable system. While the effect of tillage on soil fungal communities can vary widely (de Graaff et al., 2019), a tillage event could be expected to particularly impact on soil fungal communities, as the physical soil disturbance would negatively impact soil fungal mycelial networks (Verbruggen and Kiers, 2010), and this would appear to be the case in our study. The significant change in fungal community structure in MC between the first and the later sampling events is actually indicative of the appropriateness of our sampling strategy to pick up temporal variations in the soil microbiome, should they have occurred.

The temporal stability of the differences in fungal community structure between agricultural systems across multiple sampling events throughout the growing season is impressive, as each system undergoes considerable flux during this time period due to the application of individual management events and changing weather conditions. This is particularly in light of the fact that individual aspects of management have been shown to influence soil microbial communities, such as fertilization (Leff et al., 2015), grazing events (Du et al., 2020), and herbicide application (Druille et al., 2016).

### 4.2 Effects of agricultural system on bacterial community structure not very apparent

As was seen with soil fungal community structures, the factor ‘Time’ also had a highly significant influence on bacterial community structure (√CV = 0.042), as well as there being a significant ‘System × Time’ interaction (√CV = 0.032). Again, however, the factor ‘System’ was the far stronger determinant on bacterial community structure (√CV = 0.145), a finding which again agrees with the first hypothesis of the study. In marked contrast to that seen with fungal community structure, there were not as many differences in bacterial community structure among the different agricultural systems. This would indicate that soil fungi and bacteria do not share the same magnitude of response to differences in land management and its intensity, as has been previously suggested (Neal et al., 2021).

This finding concurs with other studies, for example a Swiss field experiment which included a grassland ley and a crop rotation of conventional and no tillage, where agricultural management system had a stronger effect on fungal community β-diversity (7%) than on bacterial β-diversity (2%, Longepierre et al., 2021). Indeed, we also previously reported the stronger influence of grassland agricultural management on soil fungal community structure, compared to bacterial community structure, at both the continental and regional scale, including the Swiss lowlands (Fox et al., 2021). In the present study, this disparity in response is even more apparent, particularly between MC and the differently managed permanent grassland systems. The system MC was persistently (i.e., over all sampling events) different in terms of its fungal community structure from each of the permanent grassland systems (INT, EXT_HP, EXT_LP and EXT_DP), but was only persistently different from EXT_HP in terms of bacterial community structure. The highly significant and persistent difference in bacterial community structure between EXT_HP and EXT_DP is most probably driven by the difference in soil pH between these two systems, as this variable is a major driver of soil bacterial communities (Lauber et al., 2009). The difference in the magnitude of the response seen with soil fungal and bacterial communities may be due to differences in the overlying plant community in the five studied agricultural systems. It has been demonstrated that plant and fungal communities are more closely associated than bacterial communities (Cassman et al., 2016), and that grassland management intensity significantly shifts plant community structure (Wesche et al., 2012).

Despite a lower number of differences in bacterial community structure between the agricultural systems being seen, three of the four significant differences seen at the first sampling event (MC ∼ EXT_HP, INT ∼ EXT_DP, EXT_HP ∼ EXT_DP) did finally persist in at least four of the following five sampling events. This is in broad agreement with the third hypothesis of this study. The significant difference in bacterial community structure between MC and EXT_DP seen at sampling event 1 did persist into sampling event 2, but not into the four subsequent sampling events. This would suggest that in this specific instance, the tillage event which occurred (after sampling event 1 and before sampling event 2) did impact the seasonal stability of this pairwise difference, as such events have been reported to do (Madegwa and Uchida, 2021). This was however the only pairwise difference, in either fungal or bacterial community structure, to be affected in this way.

### 4.3 System-specific indicator fungal OTUs persist throughout the growing season

Unique persistent indicator fungal OTUs were seen across the six sampling events, for each of the examined systems (except EXT_LP). This is further evidence in support of the second hypothesis of this study, as well as indicating the distinct microbial habitat that the different agricultural systems provide. Meanwhile for bacteria, only EXT_DP had bacterial indicator OTUs detected at each of the six sampling events, though both MC and INT did have unique indicator bacterial OTUs detected at five of the six sampling events. No persistent indicator OTUs were identified in EXT_LP. This, together with what was observed with community structure, would suggest that this system is largely indistinguishable from EXT_HP and EXT_DP, as it represents comparable conditions to both as its levels of soil P_av_ lies halfway between both.

Despite all extensive grassland receiving limited management for the past >20 years, there may be a number (or combination) of factors behind the gradient in available P between the three EXT systems. These likely include natural differences in P availability due to soil pH (Gustafsson et al., 2012), soil type or underlying bedrock, or due to a legacy layover from fertilization events which may have occurred decades previously, as has been shown for soil calcium in Swiss grasslands (Schaffner et al., 2012). The amount of time required to ‘draw-down’ soil P in agricultural soil is difficult to determine, for a plethora of reasons such as long residence times, complex chemical pathways and physicochemical processes involved in P mobilization (Cassidy et al., 2016).

The highest number of persistent fungal indicators were found in MC, despite the fact that the fungal community significantly shifted in this system as the growing season progressed due to the ploughing event between sampling events 1 and 2. The taxonomic assignments of many of these persistent fungal OTUs have been previously identified in arable systems. The fungal species *Helotiales* (fOTU_153) and the fungal genera *Chaetomium* and *Exophiala* (fOTU_227 and fOTU_2777, respectively), have also been identified in an arable field in Germany (Moll et al., 2016). While the fungal genera *Talaromyces* (fOTU_5876) and species *Plectosphaerella cucumerina* (fOTU_6397) have both been identified as biocontrol agents in arable systems, the former against nematodes (Atkins et al., 2003; Sommermann et al., 2018).

The genera *Podospora* (fOTU_837) was identified as a persistent indicator in INT, and this genera has been previously identified as an indicator for European intensively managed grasslands (Fox et al., 2021). The fact that this was found as a persistent indicator over multiple sampling events in this study does re-enforce this finding. This genera has been reported as being coprophilous (Graf and Chmura, 2006), and is probably taking advantage of the application of slurry (as fertilizer) in this system. The fungal family Clavariaceae (fOTU_703), a persistent indicator of EXT_HP, is a fungal group typical of nutrient-poor grassland (Griffith et al., 2013), and is likely benefitting from the years of extensive management in this system.

Only two persistent indicator bacterial OTUs were identified, both for the system EXT_DP. Little functional information is currently available on the bacterial family Pedosphaeraceae (Behnke et al., 2021), though they have been shown to be highly negatively correlated to soil pH in Swiss soils (Gschwend et al., 2021b). This may explain why it was identified as a persistent indicator OTU for EXT_DP, as it had the lowest soil pH of any of the study systems (pH 6.13). The genus *Pajaroellobacter* has been shown to negatively respond to the addition of N (Isobe et al., 2020), thus the lack of any nutrient additions to EXT_DP for decades may represent favorable conditions for this genus.

The results outlined in this study are indeed timely, particularly in light of recent European scale studies which examined the differences in soil fungal and bacterial community structure between differently managed grasslands (Barreiro et al., 2022; Fox et al., 2021), though these utilized only a single sampling event at the height of the growing season. The results presented herein both compliment and strengthen these studies, and demonstrate that such management induced differences are not merely present at peak plant productivity, but persist regardless of plant phenological stage, individual management events or changing weather conditions. This is an important finding in relation to informing the sampling strategy of future studies in soil microbial ecology, such as biological soil monitoring programs (i.e., the Swiss Soil Monitoring Network, Gubler et al., 2015), as the results indicate that there is a flexibility within a growing season when it comes to taking soil samples. This would greatly simplify the logistics involved in coordinating a large scale sampling campaign. Indeed, the results of this study also have important implications to the interpretation of existing studies in this discipline, the majority of which have been conducted utilizing a single sampling event.

## 5 Conclusion

Our study highlights the temporal stability of pairwise differences in soil microbiome structures. We demonstrated this between established agricultural systems, even those with a comparable management regime, through changing plant growth conditions. In particular, our study shows that such differences in fungal community structure induced by agricultural management, indeed even smaller differences, are temporally stable and can be detected regardless of sampling time throughout the growing season. The findings of this study have broader implications in relation to informing the sampling strategy of future studies in soil microbial ecology, as well as to the interpretation of the majority of the existing studies in this discipline which were conducted following a single sampling event. They would indicate that sampling campaigns for soil microbial biodiversity should focus on sampling more sites, rather than on more sampling periods throughout the growing season.

## Data accessibility

DNA sequences were deposited in the NCBI SRA archive with the project number PRJNA821020.

## Author contribution

Aaron Fox: Data curation (lead); Formal analysis (lead); Writing original draft (lead). Franco Widmer: Conceptualization (equal); Investigation (equal); Methodology (equal); Project administration (equal); Supervision (equal); Visualization (equal). Andreas Lüscher: Conceptualization (equal); Funding acquisition (lead); Investigation (equal); Methodology (equal); Project administration (equal); Supervision (equal); Visualization (equal); Writing original draft (supporting).

## Declaration of competing interest

The authors declare that they have no known competing financial interests or personal relationships that could have appeared to influence the work reported in this paper.
