# Supplementary material for: Soil microbial community structures are shaped by agricultural systems revealing little temporal variation
Source: Environ Res. 2022 Nov;214:113915. doi: 10.1016/j.envres.2022.113915 (PMC9492858; doi:10.1016/j.envres.2022.113915)
Supplement: Multimedia component 1 [file mmc1.docx]

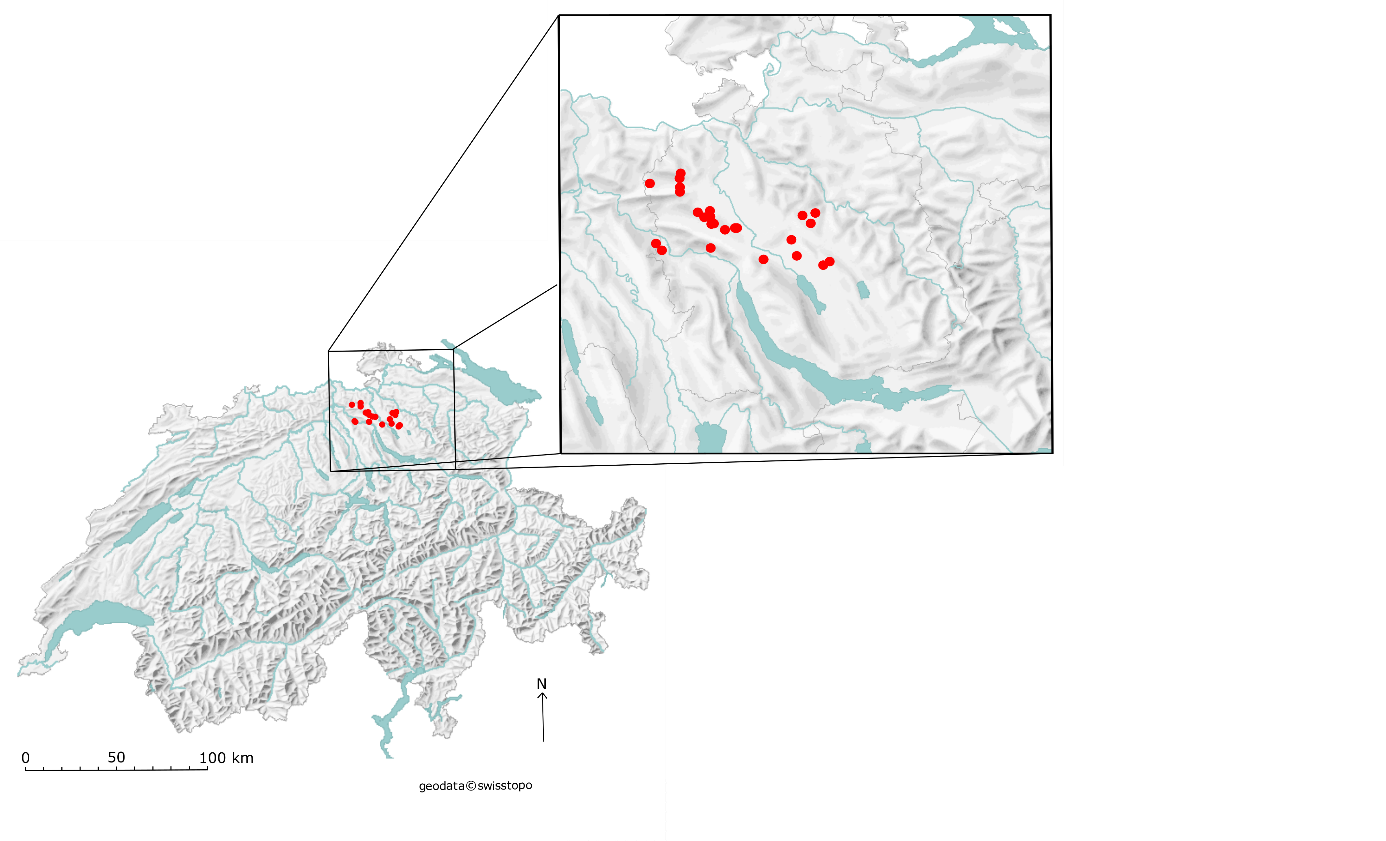


Figure S1: Graphical representation of the sampling sites in Switzerland (red dots). Sampling sites used in this study were located around the Canton of Zurich.


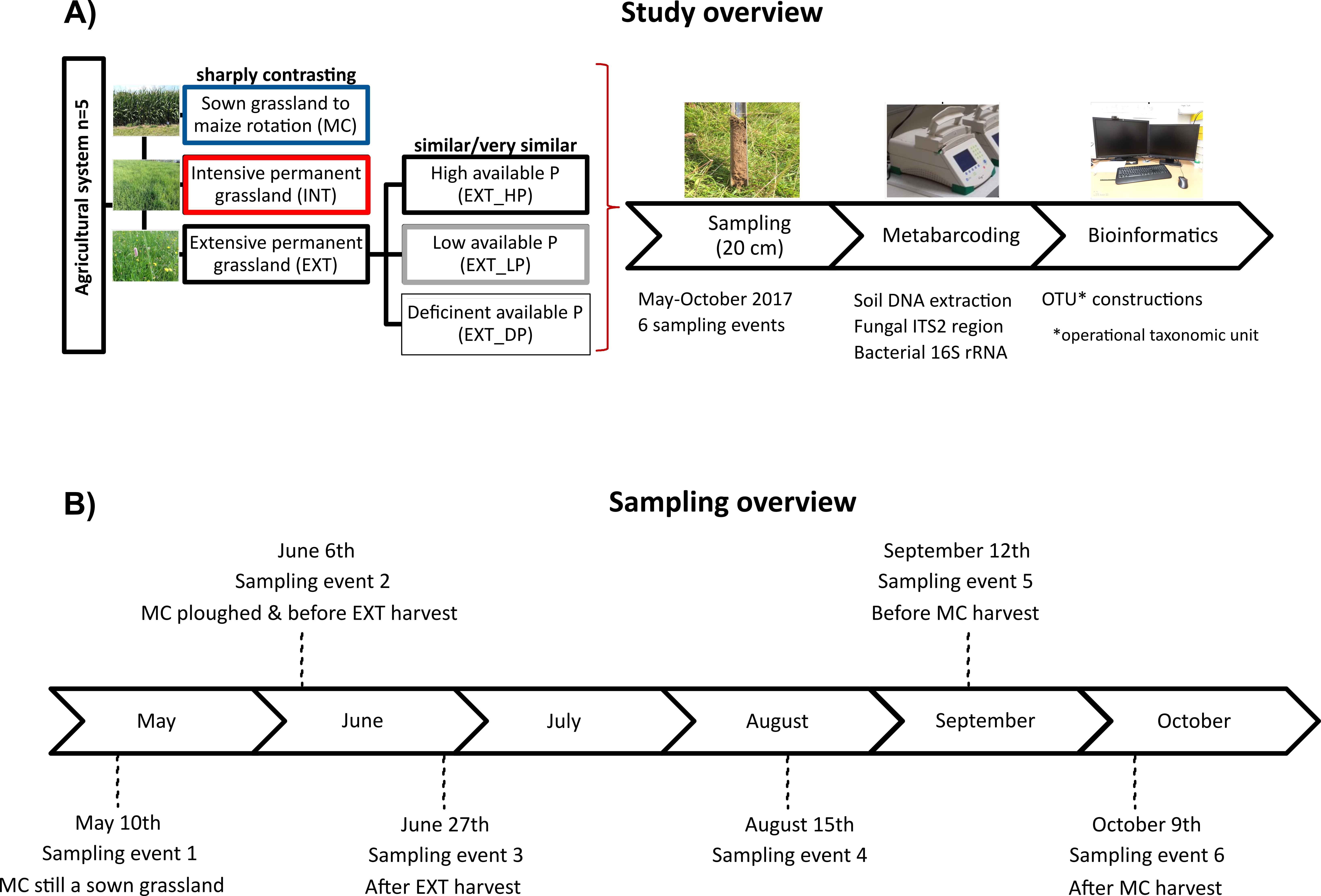


Figure S2: A graphical overview of the study work-flow (A) and sampling strategy (B). Sampling events were chosen to coincide with specific management events throughout the growing season.


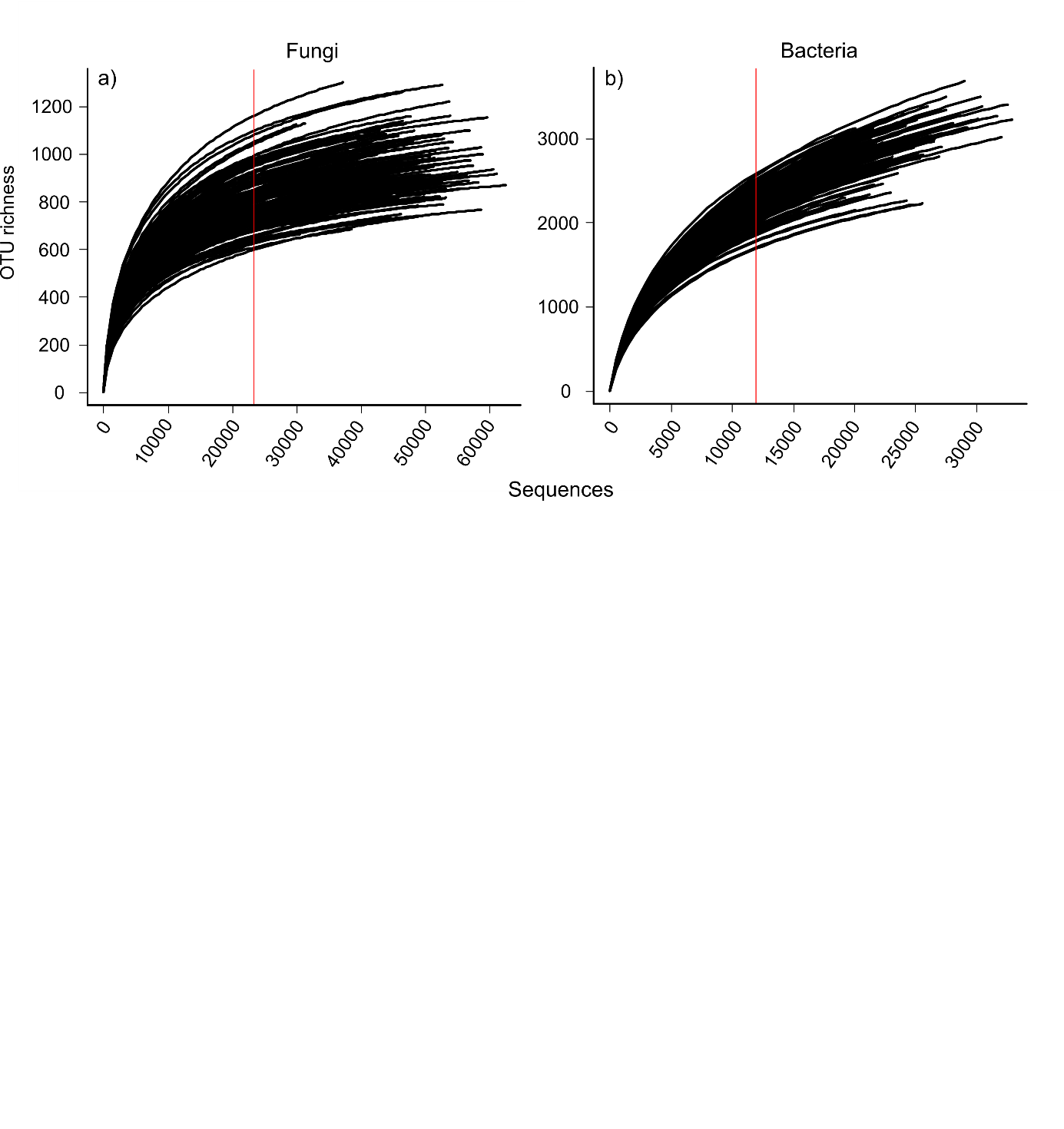


Figure S3: Rarefaction analysis displaying the sequencing depth and OTU-saturation in both the fungal (panel a) and bacterial (panel b) communities. The red lines indicate the number of high-quality sequences obtained from the sample with the lowest sequencing depth (i.e., 23,265 sequences for fungi and 11,934 sequences for bacteria).

Table S1: The influence of the factors ‘Systems’, ‘Time’ and ‘Site’ on the % rel. ab. of the major (>0.1%) fungal (a) and bacterial (b) phyla. Shown is the *F* -value and associated significance level, code: ‘***’ *P ≤* 0.001 ‘**’ *P ≤* 0.01 ‘*’ *P ≤* 0.05 ‘ns*’ P >* 0.05. Also shown is the mean (± SE) value of these measures across the four permanent grassland systems (i.e., INT, EXT_HP, EXT_MP and EXT_LP) and MC.

| (a) Fungi |  |  |  |  |  |  |  |  |
| --- | --- | --- | --- | --- | --- | --- | --- | --- |
|  | System | Time | System*Time | MC | INT | EXT_HP | EXT_LP | EXT_DP |
| Ascomycota | 14.855*** | 0.773^ns^ | 0.908^ns^ | 78.13 (±1.208) | 81.90 (±0.933) | 72.60 (±1.977) | 74.44 (±1.872) | 72.43 (±1.897) |
| Basidiomycota | 29.577*** | 0.588^ns^ | 0.884^ns^ | 15.46 (±1.245) | 9.51 (±0.791) | 22.84 (±2.060) | 20.51 (±2.032) | 22.87 (±2.141) |
| Mortierellomycota | 54.082*** | 3.712** | 1.524^ns^ | 2.42 (±0.147) | 2.99 (±0.206) | 1.13 (±0.067) | 1.47 (±0.158) | 2.04 (±0.190) |
| Mucoromycota | 4.097** | 0.515^ns^ | 1.016^ns^ | 0.31 (±0.058) | 0.27 (±0.059) | 0.12 (±0.013) | 0.13 (±0.016) | 0.18 (±0.056) |
| Glomeromycota | 13.212*** | 2.472* | 0.767^ns^ | 0.30 (±0.035) | 0.34 (±0.047) | 0.46 (±0.033) | 0.58 (±0.039) | 0.55 (±0.080) |
| Chytridiomycota | 10.900*** | 4.294** | 3.089*** | 1.02 (±0.237) | 0.58 (±0.109) | 0.44 (±0.029) | 0.27 (±0.033) | 0.21 (±0.033) |
| Rozellomycota | 24.787*** | 5.266*** | 1.162 | 0.08 (±0.010) | 1.00 (±0.161) | 0.98 (±0.107) | 0.97 (±0.259) | 0.40 (±0.113) |
| (b) Bacteria |  |  |  |  |  |  |  |  |
| Verrucomicrobia | 124.942*** | 5.507*** | 1.539^ns^ | 14.01 (±0.474) | 13.24 (±0.417) | 16.09 (±0.446) | 18.93 (±0.836) | 22.44 (±1.018) |
| Proteobacteria | 11.079*** | 55.179*** | 2.076** | 18.91 (±0.377) | 18.76 (±0.416) | 18.65 (±0.364) | 19.69 (±0.329) | 20.14 (±0.372) |
| Actinobacteria | 37.163*** | 33.450*** | 5.265*** | 19.52 (±0.569) | 19.90 (±0.649) | 19.85 (±0.765) | 18.37 (±0.558) | 15.17 (±0.537) |
| Firmicutes | 54.275*** | 8.192*** | 2.025* | 2.41 (±0.215) | 3.11 (±0.393) | 0.83 (±0.056) | 1.03 (±0.079) | 1.01 (±0.157) |
| Planctomycetes | 37.928*** | 89.364*** | 2.749*** | 11.70 (±0.407) | 12.38 (±0.424) | 12.75 (±0.369) | 12.94 (±0.426) | 14.49 (±0.315) |
| Acidobacteria | 44.589*** | 19.394*** | 2.556** | 11.81 (±0.346) | 12.98 (±0.472) | 13.04 (±0.389) | 10.86 (±0.357) | 9.77 (±0.476) |
| Chloroflexi | 35.264*** | 8.511*** | 1.947* | 13.10 (±0.451) | 12.21 (±0.210) | 11.74 (±0.207) | 10.96 (±0.435) | 9.95 (±0.396) |
| Bacteroidetes | 28.576*** | 5.672*** | 1.524^ns^ | 2.39 (±0.130) | 1.95 (±0.071) | 1.54 (±0.051) | 1.64 (±0.058) | 1.68 (±0.083) |
| Patescibacteria | 37.612*** | 9.158*** | 0.563^ns^ | 1.41 (±0.115) | 0.98 (±0.064) | 0.66 (±0.043) | 0.79 (±0.052) | 0.83 (±0.052) |
| Gemmatimonadetes | 43.264*** | 25.803*** | 2.878*** | 1.42 (±0.093) | 0.96 (±0.049) | 1.14 (±0.059) | 1.11 (±0.059) | 0.91 (±0.056) |
| Latescibacteria | 24.694*** | 6.763*** | 2.481** | 1.07 (±0.040) | 0.83 (±0.046) | 0.94 (±0.062) | 1.13 (±0.063) | 1.23 (±0.063) |
| Nitrospirae | 48.857*** | 16.724*** | 1.733* | 0.48 (±0.036) | 0.45 (±0.021) | 0.29 (±0.020) | 0.30 (±0.029) | 0.27 (±0.032) |
| Rokubacteria | 67.590*** | 2.767* | 1.099^ns^ | 0.72 (±0.031) | 1.24 (±0.090) | 1.57 (±0.055) | 1.32 (±0.085) | 1.19 (±0.068) |
| Cyanobacteria | 5.541*** | 4.647*** | 1.397^ns^ | 0.10 (±0.007) | 0.10 (±0.006) | 0.08 (±0.005) | 0.07 (±0.005) | 0.08 (±0.007) |
| Elusimicrobia | 10.363*** | 1.490^ns^ | 1.675^ns^ | 0.10 (±0.006) | 0.09 (±0.006) | 0.08 (±0.006) | 0.10 (±0.005) | 0.12 (±0.009) |
| Entotheonellaeota | 52.225*** | 2.202^ns^ | 0.599^ns^ | 0.08 (±0.013) | 0.15 (±0.015) | 0.22 (±0.015) | 0.19 (±0.015) | 0.09 (±0.012) |
| Chlamydiae | 30.334*** | 2.153^ns^ | 1.309^ns^ | 0.07 (±0.006) | 0.05 (±0.004) | 0.04 (±0.004) | 0.05 (±0.005) | 0.10 (±0.009) |

Table S2: Persistent fungal OTUs (fOTUs), which were detected as unique indicator OTUs for each of the five agricultural systems (MC, INT, EXT_HP, EXT_LP and EXT_DP), through indicator species analysis (IndVal ≥ 0.7, *P* ≤ 0.05) at 5 or 4 sampling events. The taxonomy of each detected OTU is given at the lowest known taxonomic level (down to the genus level):- k_: kingdom, p_: phylum, c_: class, o_: order, f_: family, g_: genus and s_: species

| OTU | Type | Pairwise | Taxonomy | IndVal | P - value |
| --- | --- | --- | --- | --- | --- |
| fOTU_1036 | MC | 5 | s__Scytalidium_lignicola | 0.878 | 0.003 |
| fOTU_1663 | MC | 5 | s__Arthrographis_longispora | 0.876 | 0.006 |
| fOTU_1737 | MC | 5 | s__Chytridiomycota_sp | 0.838 | 0.013 |
| fOTU_205 | MC | 5 | o__Sordariales | 0.976 | 0.001 |
| fOTU_270 | MC | 5 | p__Ascomycota | 0.817 | 0.013 |
| fOTU_2724 | MC | 5 | g__Aspergillus | 0.822 | 0.015 |
| fOTU_299 | MC | 5 | o__Pleosporales | 0.796 | 0.023 |
| fOTU_307 | MC | 5 | g__Dichotomopilus | 0.911 | 0.006 |
| fOTU_3213 | MC | 5 | k__Fungi | 0.899 | 0.002 |
| fOTU_388 | MC | 5 | s__Rhizopus_arrhizus | 0.899 | 0.009 |
| fOTU_575 | MC | 5 | s__Podospora_appendiculata | 0.808 | 0.016 |
| fOTU_935 | MC | 5 | f__Ustilaginaceae | 0.909 | 0.003 |
| fOTU_9437 | MC | 5 | g__Podospora | 0.803 | 0.015 |
| fOTU_445 | MC | 5 | s__Devriesia_sp | 0.758 | 0.038 |
| fOTU_1007 | MC | 4 | s__Stachybotrys_chartarum | 0.784 | 0.022 |
| fOTU_1145 | MC | 4 | f__Chaetomiaceae | 0.822 | 0.009 |
| fOTU_1209 | MC | 4 | s__Vermispora_fusarina | 0.750 | 0.033 |
| fOTU_168 | MC | 4 | g__Talaromyces | 0.833 | 0.013 |
| fOTU_221 | MC | 4 | s__Falciphora_oryzae | 0.857 | 0.017 |
| fOTU_265 | MC | 4 | o__Hypocreales | 0.926 | 0.003 |
| fOTU_279 | MC | 4 | s__Hypocreales_sp | 0.899 | 0.008 |
| fOTU_361 | MC | 4 | o__Helotiales | 0.930 | 0.002 |
| fOTU_399 | MC | 4 | s__Dichotomopilus_pratensis | 0.902 | 0.008 |
| fOTU_413 | MC | 4 | s__Edenia_gomezpompae | 0.942 | 0.004 |
| fOTU_4845 | MC | 4 | k__Fungi | 0.759 | 0.022 |
| fOTU_5411 | MC | 4 | s__Exophiala_sp | 0.766 | 0.004 |
| fOTU_618 | MC | 4 | o__Pleosporales | 0.840 | 0.007 |
| fOTU_668 | MC | 4 | f__Nectriaceae | 0.810 | 0.014 |
| fOTU_6951 | MC | 4 | o__Hypocreales | 0.769 | 0.022 |
| fOTU_832 | MC | 4 | s__Microascus_brevicaulis | 0.894 | 0.012 |
| fOTU_199 | INT | 5 | s__Lasiosphaeriaceae_sp | 0.872 | 0.012 |
| fOTU_230 | INT | 5 | o__Pleosporales | 0.862 | 0.007 |
| fOTU_2376 | INT | 5 | g__Plectosphaerella | 0.862 | 0.007 |
| fOTU_238 | INT | 5 | s__Valsonectria_pulchella | 0.931 | 0.001 |
| fOTU_7484 | INT | 5 | f__Sporormiaceae | 0.896 | 0.003 |
| fOTU_818 | INT | 5 | o__Hypocreales | 0.883 | 0.003 |
| fOTU_2376 | INT | 5 | g__Plectosphaerella | 0.836 | 0.014 |
| fOTU_1921 | INT | 4 | g__Saitozyma | 0.794 | 0.019 |
| fOTU_400 | INT | 4 | s__Dipodascus_geotrichum | 0.914 | 0.001 |
| fOTU_60 | INT | 4 | f__Stachybotryaceae | 0.946 | 0.004 |
| fOTU_7552 | INT | 4 | g__Mortierella | 0.801 | 0.024 |
| fOTU_8993 | INT | 4 | o__Sordariales | 0.836 | 0.010 |
| fOTU_1437 | EXT_HP | 5 | s__Branch06_sp | 0.785 | 0.025 |
| fOTU_1862 | EXT_HP | 5 | k__Fungi | 0.799 | 0.018 |
| fOTU_263 | EXT_HP | 5 | s__Pyronemataceae_sp | 0.904 | 0.008 |
| fOTU_342 | EXT_HP | 5 | p__Ascomycota | 0.923 | 0.010 |
| fOTU_4422 | EXT_HP | 5 | s__Chaetomium_nigricolor | 0.816 | 0.011 |
| fOTU_5988 | EXT_HP | 5 | f__Helotiales_fam_Incertae_sedis | 0.815 | 0.016 |
| fOTU_792 | EXT_HP | 5 | c__Agaricomycetes | 0.862 | 0.007 |
| fOTU_885 | EXT_HP | 5 | o__Chaetothyriales | 0.803 | 0.016 |
| fOTU_1509 | EXT_HP | 4 | p__Ascomycota | 0.812 | 0.017 |

Table S2 continued:

| OTU | Type | Pairwise | Taxonomy | IndVal | P - value |
| --- | --- | --- | --- | --- | --- |
| fOTU_204 | EXT_HP | 4 | s__Leucoglossum_leucosporum | 0.815 | 0.026 |
| fOTU_210 | EXT_HP | 4 | g__Hygrocybe | 0.897 | 0.021 |
| fOTU_295 | EXT_HP | 4 | s__Cuphophyllus_sp | 0.832 | 0.022 |
| fOTU_4230 | EXT_HP | 4 | c__Agaricomycetes | 0.768 | 0.019 |
| fOTU_473 | EXT_HP | 4 | k__Fungi | 0.912 | 0.002 |
| fOTU_5769 | EXT_HP | 4 | s__Herpotrichiellaceae_sp | 0.819 | 0.019 |
| fOTU_910 | EXT_HP | 4 | c__Sordariomycetes | 0.864 | 0.008 |
| fOTU_975 | EXT_HP | 4 | c__Agaricomycetes | 0.907 | 0.002 |
| fOTU_1438 | EXT_HP | 4 | o__Hypocreales | 0.811 | 0.024 |
| fOTU_1206 | EXT_DP | 5 | s__Ceratobasidiaceae_sp | 0.854 | 0.006 |
| fOTU_1580 | EXT_DP | 5 | p__Basidiomycota | 0.764 | 0.025 |
| fOTU_332 | EXT_DP | 5 | f__Hyaloscyphaceae | 0.789 | 0.023 |
| fOTU_425 | EXT_DP | 5 | s__Drechslera_sp | 0.860 | 0.010 |
| fOTU_691 | EXT_DP | 5 | s__Archaeorhizomyces_sp | 0.800 | 0.012 |
| fOTU_707 | EXT_DP | 5 | f__Chaetosphaeriaceae | 0.780 | 0.019 |
| fOTU_7679 | EXT_DP | 5 | g__Podospora | 0.858 | 0.004 |
| fOTU_907 | EXT_DP | 5 | o__Pleosporales | 0.793 | 0.017 |
| fOTU_1035 | EXT_DP | 4 | o__Pleosporales | 0.808 | 0.016 |
| fOTU_113 | EXT_DP | 4 | p__Ascomycota | 0.784 | 0.026 |
| fOTU_1278 | EXT_DP | 4 | s__Slooffia_cresolica | 0.830 | 0.011 |
| fOTU_1483 | EXT_DP | 4 | s__Lachnum_pygmaeum | 0.766 | 0.026 |
| fOTU_1561 | EXT_DP | 4 | s__Sebacinales_sp | 0.767 | 0.021 |
| fOTU_1899 | EXT_DP | 4 | s__Glomeraceae_sp | 0.745 | 0.031 |
| fOTU_2016 | EXT_DP | 4 | g__Mortierella | 0.775 | 0.021 |
| fOTU_2212 | EXT_DP | 4 | s__Orbiliomycetes_sp | 0.791 | 0.022 |
| fOTU_2519 | EXT_DP | 4 | k__Fungi | 0.781 | 0.012 |
| fOTU_352 | EXT_DP | 4 | s__Cystodendron_sp | 0.826 | 0.012 |
| fOTU_439 | EXT_DP | 4 | s__Herpotrichiellaceae_sp | 0.803 | 0.020 |
| fOTU_557 | EXT_DP | 4 | s__Herpotrichiellaceae_sp | 0.815 | 0.025 |
| fOTU_580 | EXT_DP | 4 | s__Clavaria_falcata | 0.806 | 0.021 |
| fOTU_6022 | EXT_DP | 4 | s__Sordariales_sp | 0.821 | 0.014 |
| fOTU_679 | EXT_DP | 4 | o__Agaricales | 0.797 | 0.018 |
| fOTU_806 | EXT_DP | 4 | s__Sordariales_sp | 0.848 | 0.006 |
| fOTU_904 | EXT_DP | 4 | s__GS34_sp | 0.764 | 0.021 |
| fOTU_955 | EXT_DP | 4 | c__Leotiomycetes | 0.784 | 0.017 |

Table S3: Bacterial OTUs (bOTUs), which were detected as unique indicator OTUs for each of the five agricultural systems (MC, INT, EXT_HP, EXT_LP and EXT_DP), through indicator species analysis (IndVal > 0.7, *P* ≤ 0.05) at 5 or 4 sampling events. The taxonomy of each detected OTU is given at the lowest known taxonomic level (down to the genus level):- k_: kingdom, p_: phylum, c_: class, o_: order, f_: family, g_: genus. Abbreviations are explained in the legend of Table 1.

| OTU | Type | Pairwise | Taxonomy | IndVal | P - value |
| --- | --- | --- | --- | --- | --- |
| bOTU_1106 | MC | 5 | g_Adhaeribacter | 0.848 | 0.010 |
| bOTU_1595 | MC | 5 | g_Gemmatimonas | 0.927 | 0.005 |
| bOTU_1840 | MC | 5 | g_Pirellula | 0.913 | 0.001 |
| bOTU_1998 | MC | 5 | f_Gemmataceae | 0.804 | 0.017 |
| bOTU_2605 | MC | 5 | f_Roseiflexaceae | 0.765 | 0.022 |
| bOTU_2730 | MC | 5 | c_Alphaproteobacteria | 0.824 | 0.011 |
| bOTU_5484 | MC | 5 | g_Flavisolibacter | 0.865 | 0.005 |
| bOTU_7205 | MC | 5 | f_WD2101_soil_group | 0.838 | 0.010 |
| bOTU_13351 | MC | 4 | f_Geodermatophilaceae | 0.833 | 0.011 |
| bOTU_13486 | MC | 4 | g_Gemmatimonas | 0.806 | 0.014 |
| bOTU_14608 | MC | 4 | g_Pontibacter | 0.814 | 0.014 |
| bOTU_1772 | MC | 4 | g_Flavisolibacter | 0.921 | 0.002 |
| bOTU_2104 | MC | 4 | c_Subgroup_6 | 0.777 | 0.014 |
| bOTU_2217 | MC | 4 | f_Ktedonobacteraceae | 0.772 | 0.019 |
| bOTU_2591 | MC | 4 | c_Alphaproteobacteria | 0.769 | 0.023 |
| bOTU_2681 | MC | 4 | g_Symbiobacterium | 0.776 | 0.018 |
| bOTU_2861 | MC | 4 | g_Thermomonospora | 0.741 | 0.030 |
| bOTU_2915 | MC | 4 | g_Bacillus | 0.851 | 0.008 |
| bOTU_3077 | MC | 4 | c_Sericytochromatia | 0.814 | 0.008 |
| bOTU_3084 | MC | 4 | f_Anaerolineaceae | 0.783 | 0.017 |
| bOTU_3737 | MC | 4 | f_P3OB-42 | 0.763 | 0.018 |
| bOTU_4426 | MC | 4 | g_Pontibacter | 0.805 | 0.012 |
| bOTU_4471 | MC | 4 | g_Haliangium | 0.806 | 0.010 |
| bOTU_5190 | MC | 4 | g_Ureibacillus | 0.818 | 0.013 |
| bOTU_5526 | MC | 4 | f_Gemmatimonadaceae | 0.862 | 0.003 |
| bOTU_713 | MC | 4 | o_Candidatus_Kaiserbacteria | 0.853 | 0.004 |
| bOTU_8740 | MC | 4 | g_Massilia | 0.823 | 0.015 |
| bOTU_2505 | INT | 5 | g_Bacillus | 0.853 | 0.004 |
| bOTU_6906 | INT | 5 | f_Bacillaceae | 0.910 | 0.002 |
| bOTU_1022 | INT | 4 | c_Actinobacteria | 0.835 | 0.007 |
| bOTU_2547 | INT | 4 | f_Caldilineaceae | 0.840 | 0.004 |
| bOTU_3711 | EXT_HP | 4 | g_Chthoniobacter | 0.758 | 0.024 |
| bOTU_1240 | EXT_DP | 5 | g_Gemmatimonas | 0.773 | 0.022 |
| bOTU_1564 | EXT_DP | 5 | f_Gemmataceae | 0.746 | 0.027 |
| bOTU_2208 | EXT_DP | 5 | o_Acidobacteriales | 0.768 | 0.022 |
| bOTU_2699 | EXT_DP | 5 | o_SBR1031 | 0.795 | 0.013 |
| bOTU_3017 | EXT_DP | 5 | o_MBNT15 | 0.770 | 0.019 |
| bOTU_3130 | EXT_DP | 5 | f_Ktedonobacteraceae | 0.775 | 0.022 |
| bOTU_337 | EXT_DP | 5 | g_HSB_OF53-F07 | 0.785 | 0.022 |
| bOTU_4053 | EXT_DP | 5 | f_Pedosphaeraceae | 0.811 | 0.015 |
| bOTU_530 | EXT_DP | 5 | f_Pirellulaceae | 0.831 | 0.011 |
| bOTU_766 | EXT_DP | 5 | f_WD2101_soil_group | 0.815 | 0.011 |
| bOTU_8755 | EXT_DP | 5 | g_Acidothermus | 0.752 | 0.035 |
| bOTU_996 | EXT_DP | 5 | c_OM190 | 0.744 | 0.028 |
| bOTU_1005 | EXT_DP | 4 | g_Anaeromyxobacter | 0.760 | 0.038 |
| bOTU_10212 | EXT_DP | 4 | c_BD7-11 | 0.785 | 0.021 |
| bOTU_13080 | EXT_DP | 4 | f_Xanthobacteraceae | 0.855 | 0.005 |
| bOTU_13234 | EXT_DP | 4 | g_Haliangium | 0.765 | 0.018 |
| bOTU_14105 | EXT_DP | 4 | f_Pedosphaeraceae | 0.745 | 0.029 |
| bOTU_1674 | EXT_DP | 4 | g_SM1A02 | 0.765 | 0.017 |
| bOTU_1773 | EXT_DP | 4 | f_Pedosphaeraceae | 0.846 | 0.006 |

Table S3 continued:

| OTU | Type | Pairwise | Taxonomy | IndVal | P - value |
| --- | --- | --- | --- | --- | --- |
| bOTU_1860 | EXT_DP | 4 | o_Subgroup_7 | 0.820 | 0.014 |
| bOTU_1906 | EXT_DP | 4 | d_Bacteria | 0.758 | 0.021 |
| bOTU_2231 | EXT_DP | 4 | o_JG36-TzT-191 | 0.763 | 0.013 |
| bOTU_2935 | EXT_DP | 4 | f_Phycisphaeraceae | 0.826 | 0.013 |
| bOTU_3676 | EXT_DP | 4 | f_KD3-10 | 0.762 | 0.023 |
| bOTU_4257 | EXT_DP | 4 | f_Gemmataceae | 0.785 | 0.017 |
| bOTU_446 | EXT_DP | 4 | f_Ktedonobacteraceae | 0.751 | 0.029 |
| bOTU_4694 | EXT_DP | 4 | o_S-BQ2-57_soil_group | 0.781 | 0.017 |
| bOTU_582 | EXT_DP | 4 | o_Elsterales | 0.768 | 0.025 |
| bOTU_6738 | EXT_DP | 4 | c_Subgroup_6 | 0.781 | 0.015 |
| bOTU_685 | EXT_DP | 4 | o_Subgroup_2 | 0.845 | 0.014 |
| bOTU_7360 | EXT_DP | 4 | o_Planctomycetales | 0.759 | 0.024 |
| bOTU_7391 | EXT_DP | 4 | o_MBNT15 | 0.774 | 0.023 |
| bOTU_794 | EXT_DP | 4 | f_Pedosphaeraceae | 0.751 | 0.032 |
| bOTU_800 | EXT_DP | 4 | f_WD2101_soil_group | 0.787 | 0.014 |
| bOTU_8774 | EXT_DP | 4 | f_Isosphaeraceae | 0.814 | 0.011 |
